# Supplementary material for: Increased Amyloidogenic Neuronal Injury in HIV‐1–infected APP‐KI Alzheimer's disease mice
Source: Alzheimers Dement. 2026 Sep 8;22(9):e71807. doi: 10.1002/alz.71807 (PMC13551597; doi:10.1002/alz.71807)
Supplement: Supplementary file 2 [file ALZ-22-e71807-s002.docx]

*Supplementary Materials*

**Increased Amyloidogenic Neuronal Injury**

**in HIV-1-infected APP-KI Alzheimer’s disease mice**

Shaurav Bhattarai^1^, Emma G. Foster^1^, Rana Kadry^1^, Yaman Lu, Mohit Kumar^1^, Syed Qasim^1^, Amrita Mitra^2^, Harsh Pathak^2^, Larisa Y. Poluektova^1^, Santhi Gorantla^1^, R. Lee Mosley^1^, Pravin Yeapuri^1#^, Howard E. Gendelman^1^**^#^**

*^1^Department of Pharmacology and Experimental Neuroscience, University of Nebraska Medical Center, Omaha, NE, 68106*

*^2^ Department of Pathology and Laboratory Medicine, University of Kansas Medical Center, Kansas City, KS, 66160*

**# Corresponding authors** Howard E. Gendelman, MD, Department of Pharmacology and Experimental Neuroscience, University of Nebraska Medical Center, Omaha, NE. 68198-5800; phone 402-559-4044; fax 402-559-7495; email [hegendel@unmc.edu;](mailto:hegendel@unmc.edu;) Pravin Yeapuri, Ph.D. Department of Pharmacology and Experimental Neuroscience, University of Nebraska Medical Center, Omaha, NE. 68198-5800; phone 402-559-4044; [pravin.yeapuri@unmc.edu](http://pravin.yeapuri@unmc.edu)

**Associated Data: Data Availability Statement**

All data generated, written, or analyzed from this study were included in the submitted article or its supplementary files.

 **Supplementary Figure 1**

**Supplementary Figure 1: Human microglia-like cell expression in hIL34 and hNAIL mice**. Representative IHC staining of 5 μm thick sagittal brain section from 6-months old NOG, hIL34+HIV-1, hNAIL, and hNAIL+HIV-1, 8 weeks post HIV-1 infection mice showing expression of human microglia markers P2RY12 and TMEM119 using human-specific antibodies. Inset regions at higher magnification highlight the morphology and expression of human microglia-like cells. Scale bars: main image = 200 μm; insets =50 μm

**Supplementary Figure 2**

**Supplementary Figure 2. Validation of amyloid fibril formation in intraneuronal human Aβ** (A) Representative IHC staining of 5 μm thick sagittal brain sections from 6-months-old NOG, hIL34+HIV-1, hNAIL, and hNAIL+HIV-1 mice collected 8 weeks post-infection, showing amyloid fibril-positive intraneuronal human amyloid deposition in cortex and hippocampus. Insets highlight regions of amyloid accumulation. Scale bars: main image = 200 μm; insets =50 μm.

**Supplementary Figure 3**

**Supplementary Figure 3. Validation of β-sheet amyloid formation in intraneuronal human Aβ** (A) Representative IHC staining of 5 μm thick sagittal brain sections from 6-months-old NOG, hIL34+HIV-1, hNAIL, and hNAIL+HIV-1 mice collected 8 weeks post-infection, showing Thioflavin T positive intraneuronal human amyloid deposition in cortex and hippocampus. Insets highlight regions of amyloid accumulation. Scale bars: main image = 200 μm; insets =10 μm.

**Supplementary Figure 4**

**Supplementary Figure 4. Validation of human Aβ deposition using Aβ-specific 82E1 antibody** (A). Representative IHC staining of 5 μm thick sagittal brain sections from 6-months-old NOG, hIL34+HIV-1, hNAIL, and hNAIL+HIV-1 mice collected 8 weeks post-infection, using Aβ-specific monoclonal antibody 82E1 in the cortex and hippocampus. Insets highlight regions of amyloid accumulation. Scale bars: main image = 200 μm; insets = 50 μm.

**Supplementary Figure 5**

**Supplementary Figure 5: Histopathology-guided ROI selection.** Representative IHC staining of adjacent brain sections used for ROI selection showing regions of (A) Single pathology, HIV-1 only and Aβ only, and (B) Dual pathology, Aβ+HIV-1 pathology. Insets show magnified ROIs. Scale bars: main image = 200 μm, insets = 50 μm. (C) Representative whole-brain IF image showing the spatial distribution of selected ROIs used for GeoMx DSP analysis.

**Supplementary Table 1: Raw plasma viral load**

| **Treatment group** | **hIL34 + HIV-1** | | **hNAIL + HIV-1** | |
| --- | --- | --- | --- | --- |
| **Weeks post infection** | **2 weeks** | **8 weeks** | **2 weeks** | **8 weeks** |
| **Viral load (copies/mL)** | 1080 | 136000 | 16000 | 285000 |
|  | 62700 | 488000 | 815 | 462000 |
|  | 4310 | 391000 | 23200 | 461000 |
|  | 449000 | 213000 | 2690 | 179000 |
|  | 1520 | 194000 | 10200 | 216000 |
|  | 270000 | 1500000 | 8040 | 347000 |
|  | 51800 | 383000 | 1770 | 20700 |
|  | 107000 | 131000 | 1130 | 214000 |
|  | 1490 | 402000 | 1290 | 132000 |
